# Steatotic Liver Disease and Sepsis Outcomes—A Prospective Cohort Study (SepsisFAT)

**DOI:** 10.3390/jcm13030798

**Published:** 2024-01-30

**Authors:** Juraj Krznaric, Neven Papic, Nina Vrsaljko, Branimir Gjurasin, Marko Kutlesa, Adriana Vince

**Affiliations:** 1 Department of Infectology, School of Medicine, University of Zagreb, 10000 Zagreb, Croatia; jkrznaric@bfm.hr (J.K.); mkutlesa@bfm.hr (M.K.); avince@bfm.hr (A.V.); 2 Department for Adult Intensive Care and Neuroinfections, University Hospital for Infectious Diseases Zagreb, 10000 Zagreb, Croatia; nvrsaljko@bfm.hr (N.V.); bgjurasin@bfm.hr (B.G.); 3 Department for Viral Hepatitis, University Hospital for Infectious Diseases Zagreb, 10000 Zagreb, Croatia

**Keywords:** steatotic liver disease, SLD, NAFLD, sepsis, community-acquired infections, metabolic syndrome

## Abstract

**Background**: While it has been shown that steatotic liver disease (SLD) is associated with systemic changes in immune response, the impact of SLD on sepsis outcomes has not yet been established. The aim of this study was to investigate the association between SLD and sepsis severity and outcomes. **Methods**: A prospective observational study included consecutively hospitalized adult patients with community-acquired sepsis during a 16-month period. **Results**: Of the 378 included patients (49.5% male, median age of 69, IQR 57–78 years), 174 (46%) were diagnosed with SLD. Patients with SLD were older and more frequently fulfilled the criteria for metabolic syndrome. There were no differences in the source and etiology of sepsis between the groups. Patients with SLD exhibited a higher incidence of acute kidney injury (29.3% vs. 17.6%), the need for renal replacement therapy (16.1% vs. 8.8%), and more frequent use of invasive mechanical ventilation (29.3% vs. 18.1%). In-hospital mortality was significantly higher in the SLD group (18.39% vs. 9.8%). The multivariable analysis indicated that SLD was associated with mortality (HR 2.82, 95% CI 1.40–5.71) irrespective of the other elements within metabolic syndrome. **Conclusions**: SLD might be associated with higher sepsis in-hospital mortality, and more frequent development of acute kidney and respiratory insufficiency requiring more critical care support.

## 1. Introduction

Sepsis is a life-threatening organ dysfunction caused by a dysregulated host response to infection, often resulting in severe multiorgan failure with a high mortality rate of 25–30% [1,2,3]. According to the Sepsis-3 consensus definition, organ dysfunction is defined as an acute change in a total sequential (sepsis-related) organ failure assessment (SOFA) score ≥ 2 points consequent to the infection [1]. The liver is a critical organ for host survival in sepsis, and a balanced liver-derived pro- and anti-inflammatory response is crucial for bacterial clearance and the resolution of inflammation [4]. Although rare, liver failure in sepsis significantly increases mortality, surpassing acute renal or respiratory insufficiency [4]. Chronic liver dysfunction also contributes to the risk of an infection progressing to sepsis and impacts survival [5,6].

Steatotic liver disease (SLD), until recently known as non-alcoholic fatty liver disease (NAFLD), is the most widespread chronic liver condition, impacting around 30% of the world’s population, with a vast majority of cases still undiagnosed [7,8,9]. SLD is linked with chronic low-grade inflammation, microvascular endothelial dysfunction, insulin resistance and impaired immune responses, all of which might have a profound effect on the course of sepsis and its outcomes [10,11,12].

While other components of metabolic syndrome (MetS) linked with SLD, specifically T2DM and obesity, have been extensively studied as risk and prognostic factors, often with conflicting results, there are only a few minor retrospective studies that included SLD as a variable and examined its impact on infection outcomes. According to limited data, SLD is associated with higher mortality and adverse outcomes in community-acquired pneumonia, bacteriemia of gastrointestinal origin, more frequent development of *Clostridioides difficile*-associated diarrhea in the elderly population and recurrent urinary tract infections in premenopausal women [13,14,15,16,17,18]. In COVID-19, SLD might be associated with increased susceptibility to SARS-CoV-2 infection, the need for hospitalization, and complications including pulmonary thromboembolism; nevertheless, the influence of SLD on COVID-19 mortality remains uncertain [19,20].

To the best of our knowledge, there are no prospective studies analyzing the impact of SLD on the course of sepsis and its outcomes. Therefore, the role of SLD in severe infections is still unclear. Here, we conducted a prospective cohort study to investigate an association between SLD and sepsis severity and its outcomes.

## 2. Materials and Methods

### 2.1. Study Design and Patients

Between January 2022 and May 2023, a prospective observational, non-interventional study enrolled adult patients consecutively admitted to the University Hospital for Infectious Diseases Zagreb (UHID) due to severe community-acquired bacterial infections (Project SepsisFAT, Clinical Study Identifier NCT06021743). Upon admission, patients were screened for sepsis and were included in the study if there was a clinical suspicion of sepsis, and if they met Sepsis-3 criteria [1]. The clinical characterization of patients with sepsis was defined as organ dysfunction measured with an acute change in total SOFA score ≥ 2 points consequent to the infection [1].

The predetermined exclusion criteria were: individuals under the age of 18, the lack of written informed consent, immunosuppression, malignancies, autoimmune diseases, pregnancy, HIV, chronic viral hepatitis, and the presence of other chronic liver conditions such as hemochromatosis, Wilson’s disease, toxic hepatitis, deficiency of alpha-1-antitrypsin, and autoimmune liver disease. Excessive alcohol consumption was defined as an average intake exceeding >21 standard drinks per week for men or >14 standard drinks per week for women, based on US standards [21].

For this study, we exclusively included patients with community-acquired infections. Community-acquired infections are defined as infectious diseases acquired outside of healthcare or hospital settings. Furthermore, patients with recent COVID-19 infections (within 3 months) were not included in the study. Additionally, if participants contracted COVID-19 during their hospital stay, they were excluded.

**Figure 1:**
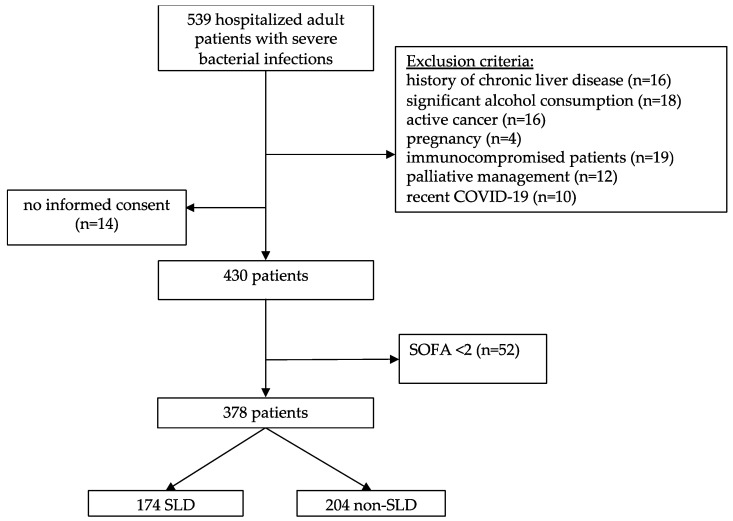
Study design flow chart.

During the study period, a total of 539 consecutively hospitalized adult patients with a presumed bacterial infection were screened. A total of 109 patients were eliminated from the study due to exclusion criteria, as described in Figure 1. Of the remaining 430 patients, 52 had a SOFA score < 2, and finally, 378 patients were included in the study. Patients who entered the study were screened for the presence of components of MetS and SLD using non-invasive procedures (ultrasound, controlled attenuation parameter—CAP, transient elastography), as described below. The study adhered to the ethical principles outlined in the Declaration of Helsinki and received approval from the Ethical Committee of the University Hospital for Infectious Diseases (UHID) in Zagreb, Croatia (protocol code 01-1247-2-2019 and approval granted on 30 August 2019). According to the power analysis, the enrolment of 360 patients was required to achieve an 80% chance of detecting a 10% difference in mortality between the two groups (SLD vs. non-SLD) at a 5% significance level.

### 2.2. Data Collection, Definitions and Outcomes

Routine demographic, clinical, microbiological, treatment and laboratory parameters were collected in a standardized form, and patients were followed until discharge. Anthropometric measurements including body mass index (BMI), waist circumference (WC), waist-hip ratio (WHR) and waist-height ratio (WHtR) were measured in all patients upon enrolment. Routine laboratory tests were collected upon admission: C-reactive protein (CRP), procalcitonin, lactate, bilirubin, aspartate aminotransferase (AST), alanine aminotransferase (ALT), gamma-glutamyl transferase (GGT), alkaline phosphatase (ALP), bilirubin, serum albumin concentration, white-blood-cell count (WBC), neutrophil-to-lymphocyte ratio, hemoglobin, blood urea nitrogen (BUN), serum creatinine, platelet count, glucose, prothrombin time/INR, fibrinogen, albumins, triglycerides (TG), cholesterol, high-density lipoprotein (HDL), and low-density lipoprotein (LDL), and urine analysis. Microbiological data included: the results of urine, blood, CSF and/or other cultures; antimicrobial susceptibility results; and the molecular detection of a specific pathogen. Disease severity scores were calculated using APACHE II and SOFA scores. APRI, FIB-4, NAFLD and FAST scores were calculated as surrogate markers of liver fibrosis and inflammation.

Steatosis severity was assessed by CAP, a method that grades steatosis by measuring the extent of ultrasound attenuation caused by hepatic fat. This method relies on simultaneous transient elastography (TE), which estimates the fibrosis levels. CAP has been extensively studied and showed high AUC of 0.82 and 0.88 in diagnosing the presence of steatosis and stage III steatosis, respectively. Various studies have proposed different CAP cut-off values corresponding to specific grades of liver steatosis as defined by biopsy, and in this study, we used the following grading system: grade I CAP > 250 dB/m (S1 ≥ 10% of hepatocytes with fat accumulation), grade II CAP > 280 dB/m (S2 ≥ 33% of hepatocytes with fat accumulation) and grade III CAP > 300 dB/m (S2 ≥ 66% of hepatocytes with fat accumulation) [22,23,24]. TE was employed to assess the fibrosis stage, demonstrating a high AUC in predicting both the stage of fibrosis and the presence of cirrhosis in non-septic patients (with an AUC of 0.84 for F2 fibrosis and 0.93 for F4 fibrosis) [25]. Patients were also screened for the components of MetS, according to IDF criteria, including insulin resistance, abdominal obesity, high blood pressure, and dyslipidemia, which involves decreased levels of HDL cholesterol, and elevated levels of TG [26].

Although the nomenclature of NAFLD was changed during the period studies, we used definition valid at time point when study started [9,27]. Therefore, the study excluded patients with alternative causes of liver steatosis, concurrent liver conditions, and/or any of the exclusion criteria mentioned above. Following the study protocol, all patients underwent testing for HIV, HBsAg, and anti-HCV upon admission. The main outcome assessed in the study was in-hospital mortality. Secondary outcomes encompassed the occurrence of septic shock, organ dysfunction (evaluated through the SOFA score, involving acute kidney injury, acute liver failure, respiratory failure, and encephalopathy), ICU admission (involving the requirement and duration of mechanical ventilation, renal replacement therapy, vasopressor therapy), length of hospital stay, and hospital-acquired infections.

### 2.3. Statistical Analysis

Clinical characteristics, laboratory and demographic data were evaluated and presented descriptively. The Shapiro–Wilk test was used to check for normality of continuous variables’ distribution. Fisher’s exact test and Mann–Whitney U test was used to compare the two groups. Survival analysis was evaluated using the Kaplan–Meier method, and the comparison between groups was made using the log-rank test. Risk factors associated with negative outcomes were investigated using a univariate, and subsequently a multivariable, Cox regression model by estimating the hazard ratio (HR) and its 95% confidence intervals (95% CI). Statistical analyses were performed using GraphPad Prism Software, version 10 (San Diego, CA, USA).

## 3. Results

### 3.1. Baseline Patient’s Characteristics

Overall, 378 patients were included in the study (187; 49.47% male), with a median age of 69 [IQR 57–78] years. SLD was diagnosed in 174 patients (46.03%), of which 92 (52.87%) were male. Table 1 displays the baseline distinctions between the groups. Patients with SLD were older, more frequently had T2DM, arterial hypertension, dyslipidemia, and COPD, more frequently fulfilled criteria for MetS, and more commonly had one or more components of MetS (all 5 components were present in 18, 10.34% vs. 3, 1.47%; 4 components in 37, 21.26% vs. 13, 6.37%). The median Charlson comorbidity index (CCI) was higher in the SLD group (4 [IQR 2–5.8] vs. 3 [IQR 1–5]).

On baseline evaluation, the SLD group had a significantly higher CAP value (median 300 dB/m [IQR 273–335] vs. 202 dB/m [IQR 170–225]); 87 (50.0%) had grade 3, 33 (18.97%) grade 2 and 54 (31.03%) grade 1 steatosis. In addition, the SLD group had higher TE values (6.2 kPa [IQR 5.1–9] vs. 5.2 kPa [IQR 4–8.1]), as well as calculated APRI, FIB-4, NAFLD and FAST scores. There was a significant difference in the NAFLD fibrosis score (0.43 [IQR −0.62–2.1] vs. −0.48 [IQR −1.9–1.1] *p* < 0.0001)), FIB-4 (2.1 [IQR 1.2–3.4] vs. 1.6 [IQR 0.91–2.7] *p* = 0.0056) and FAST score (0.31 [IQR 0.15–0.52] vs. 0.1 [IQR 0.041–0.27], *p* = <0.0001). 77 (44.25%) patients with NAFLD had a FAST score > 0.35 in contrast to 39 (19.12%) patients without NAFLD (*p* < 0.0001).

The duration from symptom onset to hospital admission was comparable in both groups, with a median of 4 (IQR 2–6) days. Similarly, the severity of the disease upon hospital admission, assessed by SOFA and APACHE II scores, was alike between the two groups (refer to Table 1).

There were no differences in infection origin between two groups, with lower-respiratory tract infections being the most common (pneumonia; 39, 22.41% vs. 48, 23.41%), followed by skin (18.39% vs. 10.24%), gastrointestinal (GI; 16.09% vs. 13.66%) and urinary tract infections (UTI; 14.94% vs. 21.95%).

Overall, the etiology was identified in 204 (53.97%) of patients. One hundred and four (104, 27.51%) had positive blood cultures (SLD 50, 28.74% vs. non-SLD 54, 26.47%) with the most common isolate being *E. coli* (11, 22% vs. 13, 24.07%), followed by *S. aureus* (MSSA) (16.00% vs. 12.96%) and *S. pneumoniae* (12.00% vs. 16.67%). A total of 88 (50.57%) patients in the SLD group and 86 (42.16%) patients in the non-SLD group did not have an isolated causative agent of the disease (Supplementary Table S1).

Regarding laboratory findings upon admission, there was no statistical significance in routine inflammatory markers, including CRP (206 mg/L, (118–291) vs. 198 mg/L, (88–280)), procalcitonin, lactate (2.0 mmol/L (1.2–3.1) vs. 1.9 mmol/L (1.3–3.1) and WBC, as shown in Supplementary Table S2. Patients with SLD had higher AST (33 U/L (23–60) vs. 26 U/L (18–48), *p* = 0.0002), ALT (33 U/L (19–55) vs. 24 U/L (15–45), *p* = 0.0069) and GGT levels, as well as the admission blood glucose (7.6 mmol/L (6.5–9.8) vs. 6.7 mmol/L (5.8–8.1), *p* < 0.0001) and TG (1.9 mmol/L (1.4–2.9) vs. 1.4 mmol/L (1.1–2), *p* < 0.0001), and lower eGFR (64 mL/min/1.73 m^2^ (36–86) vs. 69 mL/min/1.73 m^2^ (45–94), *p* = 0.0314) than the non-SLD group. There were no significant differences in other routine laboratory findings, as shown in Supplementary Table S2. 

### 3.2. Clinical Course and Outcome of Sepsis

There were no differences in the length of hospital stay, need for ICU admission, or the need for and duration of vasopressor treatment between the groups, as shown in Table 2. However, patients with SLD more frequently required invasive mechanical ventilation (OR 1.87, 95% CI 1.16–2.99), more frequently developed acute kidney injury (OR 1.67, 95% CI 1.02–2.73) and required renal replacement therapy (OR 1.98, 95% CI 1.08–3.66). The rates of nosocomial infections and cardiac complications were similar between groups.

During hospitalization, 52 (13.75%) patients died, of which 24 (46.15%) were male. In-hospital mortality was significantly higher in the SLD group (32, 18.39% vs. 20, 9.8%). Furthermore, early (within 48 h), 7- and 28-day mortality was significantly higher in the SLD group (Table 3). Time from hospital admission to death was significantly shorter in the SLD group (7 (4–17) vs. 18 (10–28) days, *p* = 0.0020). In a subgroup analysis of patients admitted to the ICU, patients with SLD had significantly higher ICU mortality (28, 43.75% vs. 16, 25.81%, OR 2.23, 95% CI 1.02–4.77, *p* = 0.0347).

### 3.3. Factors Associated with Mortality

Next, we examined the factors contributing to in-hospital mortality. The differences in baseline clinical, laboratory, and microbiological characteristics between survivors and non-survivors are shown in Supplementary Table S3.

In the univariable analysis, there were no differences in age and sex, BMI, obesity, waist circumference, presence of arterial hypertension and other comorbidities, except for T2DM and SLD between survivors and non-survivors, as shown in Table 3. Regarding laboratory findings upon admission, procalcitonin ≥ 3.0 µg/L, lactate ≥ 2.5 mmol/L, eGFR ≤ 50 mL/min/1.73 m^2^, LDH ≥ 260 IU/L, albumin ≤ 30 g/L, INR ≥ 1.3, D-dimer ≥ 4.0 mg/L, cholesterol ≤ 2.8 mmol/L, TG ≤ 1.7 mmol/L, and HDL ≤ 0.6 mmol/L were associated with higher mortality. Regarding liver-related scores, higher mortality was associated with CAP ≥ 270 dB/m, liver stiffness ≥ 6.6 kPa, NAFLD fibrosis score ≥ 1.3, FIB-4 score ≥ 3.0, and FAST score ≥ 0.35. Mortality was higher among patients with respiratory and non-urinary tract infections, patients with higher SOFA and APACHE II scores, as well as in those treated at the ICU, with ARDS, acute renal failure, nosocomial infections, and those requiring mechanical ventilation (Table 3).

**Figure 2:**
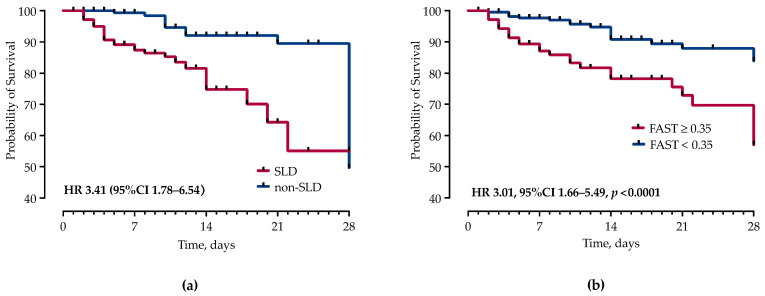
Kaplan–Meier curves and Cox proportional hazard ratios (HR) with corresponding 95% confidence intervals (95% CI) for the probability of 28-day survival stratified by presence of: (**a**) SLD; and (**b**) FAST score.

However, in the multivariable Cox proportional hazards regression analysis, after adjustment for potential confounders, SOFA ≥ 5 (HR 2.61, 95% CI 1.49–4.58), SLD (HR 2.82, 95% CI 1.40–5.71), admission lactate ≥ 2.5 mmol/L, INR ≥ 1.3 (HR 2.59, 95% CI 1.13–6.25), FAST score ≥ 0.35 (HR 2.24, 95% CI 1.24–4.12), non-UTI source (HR 1.53, 95% CI 1.04–2.22), acute renal insufficiency (HR 5.58, 95% CI 2.68–12.02), IMV (HR 3.95, 95% CI 1.06–13.85) and nosocomial infections (HR 3.84, 95% CI 1.50–10.58) remained independently associated with higher mortality, as shown in Table 3 and Figure 2. None of the other comorbidities, including components of MetS, were associated with mortality in our model. The multivariable model included age and variables that were significantly associated with mortality in univariable analyses, except for creatinine (which is already included in eGFR), respiratory source (included in non-urinary tract source), CAP and liver stiffness (included in the FAST score). The area under the ROC curve in the fully adjusted model was AUC 0.89 (95% CI 0.86 to 0.94).

## 4. Discussion

In this observational prospective study, we provide the first evidence that SLD is associated with the course of sepsis and its outcomes. Patients diagnosed with SLD exhibited significantly higher in-hospital mortality rates and an increased need for IMV, elevated rates of acute kidney injury and the need for renal replacement therapy. Moreover, this was independent of other components of MetS. These findings point to the importance of SLD as a potential risk factor in severe bacterial infections and might have implications for clinical management and future studies for several reasons.

First, we found a high prevalence of SLD and other components of MetS in hospitalized patients with sepsis. While prevalence in the general European population is estimated at around 25% [7], in our cohort, 46% of patients were diagnosed with SLD, suggesting an association between SLD and the risk of hospitalization. While overrepresentation of SLD has been reported in hospitalized COVID-19 patients [19,28], there are no reports on SLD prevalence in hospitalized patients with other community-acquired infections.

In a recently conducted population-based cohort study in Sweden, individuals with biopsy-confirmed SLD were found to be at a markedly elevated risk of severe infections necessitating hospitalization in comparison to the general population. Notably, this heightened risk was evident even in cases of simple steatosis and further increased with the severity of the disease [29]. The risk of sepsis more than doubled in patients with SLD [29].

Next, SLD should be viewed in the context of MetS. Expectedly, SLD was associated with components of MetS in our cohort. Patients with T2DM face a two-fold increased risk of developing SLD, and reciprocally, patients with SLD have a heightened risk of T2DM, indicating a bidirectional relationship mainly driven by the insulin resistance that has a central role in the pathogenesis of both conditions [30,31]. Notably, both SLD and T2DM independently elevate the risk of cardiovascular disease (CVD), with those having both conditions considered at a particularly high risk [32]. Consequently, patients with both T2DM and SLD may benefit from more intensive cardiovascular prevention strategies, and certain anti-diabetic therapies have shown positive effects on both SLD and CVD [31,32,33].

Both T2DM and obesity were extensively studied as risk factors for sepsis outcomes, often with conflicting results. While early reports suggested that T2DM is associated with increased sepsis mortality [34,35], a recent meta-analysis encompassing 21 studies showed that, rather than T2DM, high admission glucose levels, irrespective of diabetes status, were associated with an increased risk of in-hospital mortality [36]. Similarly, a large Chinese nationwide population-based cohort study showed that patients with T2DM more frequently develop acute kidney injury (AKI) and require renal replacement therapy (RRT), but this was not associated with mortality [37]. Conversely, sepsis survivors with pre-existing diabetes had a higher long-term risk of major cardiovascular events and increased mortality [38].

The data on the impact of obesity on sepsis survival are even more controversial. According to an updated systemic review including 105,159 patients, BMI > 25.0 kg/m^2^ was associated with reduced short-term mortality of patients with sepsis or septic shock [39]. However, these data should be interpreted with caution, as was recently shown in a large US cohort of patients with sepsis where an “obesity paradox” was not observed after adjustment for illness severity (APACHE 3 score, lactate levels or mean arterial pressure) [40]. Notably, none of these studies included SLD as a variable.

Furthermore, we found that of all components of MetS, only SLD was associated with mortality. There are only a few studies that reported the impact of SLD on sepsis or bacterial infections.

In a recently published case–control study involving 250 patients admitted to a general ICU, individuals with SLD exhibited higher ICU mortality rates (64% vs. 31%), a higher incidence of sepsis and septic shock, and increased requirements for critical care when compared to those without SLD [41]. ICU mortality was correlated with FIB-4 and NAFLD fibrosis scores in the SLD group [41]. Similarly, patients with SLD in our cohort more frequently developed AKI and required RRT and IMV. Two retrospective studies examined the impact of SLD on community-acquired pneumonia survival. In a cohort of hospitalized patients with non-severe community-acquired pneumonia, the mortality rate was significantly higher in the SLD group (17% vs. 5.8%) and in a multivariate analysis, even SLD without significant fibrosis was independently associated with mortality regardless of the other components of metabolic syndrome [14]. In a Croatian retrospective cohort including only patients with severe community-acquired pneumonia treated in the ICU, SLD was an independent predictor of mortality (50% vs. 20.7%) [13]. Patients with SLD more frequently developed ARDS, required IMV, respiratory ECMO and CRRT; these were consistent in all etiology groups [13]. Other studies suggested associations between SLD and recurrent urinary tract infections, *Clostridioides difficile*-associated disease, and primary bacteremia of gastrointestinal origin [15,16,17,18]. However, the studies on this topic are limited, retrospective, with a small number of patients included, and coexisting components of MetS and stages of liver disease were not consistently specified or considered. Therefore, our study recognizes the importance of SLD as an autonomous risk factor in severe bacterial infections, highlighting its potential effects on sepsis outcomes.

In addition to other well-established risk factors for sepsis mortality, such as acute kidney injury, requirement of CRRT, SOFA score, which all reflect more severe forms of the disease, we identified liver-related scores, specifically the FAST score, as an independent predictor of mortality. The FAST score was recently developed to non-invasively identify patients at risk of progressive non-alcoholic steatohepatitis, and encompasses liver stiffness, and CAP and AST levels [42]. Interestingly, fibroelastography-based scores have not been investigated as prognostic markers in severe infections. Few reports showed a FAST score > 0.35 as an independent risk factor for 30-day mortality or the need for IMV in COVID-19 patients [43]. While it could reflect the severity of sepsis due to the AST and LS increase, APRI, FIB-4, NAFLD and FAST scores were elevated in patients with liver steatosis irrespectively of other findings, suggesting a more pronounced liver injury in this patient group. This could indicate that mortality further increases in patients with more advanced liver disease. The integration of liver-injury parameters, such as the FAST score, into existing sepsis prognostic models may enhance their predictive accuracy, enabling clinicians to identify high-risk patients earlier and tailor management strategies accordingly.

Several factors may connect SLD with infections. The liver plays a significant role in the elimination of bacteria and toxins from the bloodstream and produces acute-phase proteins that contribute to the systemic activation of the immune response and the antimicrobial capabilities of the complement system [4]. However, in patients with SLD this balanced liver immune response appears to be disrupted. Patients with SLD tend to have increased levels of inflammatory cytokines, chemokines, complement and iron-binding proteins (such as CRP, IL-1β, IL-6, TNF-α, and ICAM-1) [12]. Furthermore, the complex pathogenesis of SLD includes gut dysbiosis, insulin resistance, metabolic and fat tissue dysfunction, as well as changes in cellular immunity, the neutrophil-to-lymphocyte ratio, and dysregulation of CD4 T cell function [10,11,44]. However, the impact of persistent low-level inflammation on the systemic response to infection remains unclear. We can hypothesize that patients with SLD might respond differently to infections, which could influence their outcomes. This has recently been suggested in COVID-19, where patients with SLD had distinct cytokine profiles that were associated with adverse outcomes [45,46].

Our study should be viewed within its limitations. We have included only patients with community-acquired infections, therefore these results might not reflect the impact of SLD in other clinical settings. As per the study design, we recruited patients outside of the ICU if they had SOFA ≥ 2 (66% of our cohort), which might be a selection bias that reflects in lower disease severity and consequently lower mortality than that reported in other studies. Most patients were unaware of having SLD, and data on SLD severity before current infection episodes were unavailable, thus limiting the analysis of preexisting SLD severity on patient outcomes. Similarly, liver stiffness measurements might reflect the severity of sepsis but not the stage of liver disease. Long-term follow-up after hospital discharge was not performed, which might potentially result in an underestimation of late complications after hospital discharge.

Nevertheless, we report results in a well-defined cohort of patients and provide the first prospective data on SLD impact on severe bacterial infections and sepsis outcomes. To gain a more comprehensive understanding of the role of SLD in infections, larger longitudinal studies encompassing a more diverse population are warranted.

## 5. Conclusions

In conclusion, this prospective cohort study reports on the significant impact of SLD on the course of sepsis and its outcomes. SLD was associated with increased in-hospital sepsis mortality, irrespective of the other components of metabolic syndrome. Furthermore, patients with SLD more frequently developed complications and required more critical care support. This highlights the need to consider SLD as a variable in future research on sepsis outcomes and treatment strategies. Thus, modulating liver function and the liver immune response during sepsis could be a novel strategy for regulating systemic immune responses and preventing multiple organ dysfunction.

## Figures and Tables

**Table 1:** Baseline patients’ characteristics.

	SLD (*n* = 174)	Non-SLD (*n* = 204)	*p*-Value *
Age, years, median (IQR)	69 (57–78)	64 (50–77)	0.0156
Male sex, *n* (%)	92 (52.87%)	95 (46.57%)	0.2563
Body mass index, kg/m^2^	30 (27–36)	25 (22–28)	<0.0001
Waist-hip ratio	1.0 (0.94–1.0)	0.95 (0.84–1.0)	<0.0001
Waist-height ratio	1.7 (1.5–1.9)	2.0 (1.8–2.2)	<0.0001
Controlled Attenuation Parameter (dB/m)	300 (273–335)	202 (170–225)	<0.0001
Liver stiffness (kPa)	6.2 (5.1–9)	5.2 (4–8.1)	0.0001
Smoking	37 (21.26%)	38 (18.63%)	0.6050
Moderate alcohol consumption	22 (12.64%)	18 (8.82%)	0.2442
Charlson comorbidity index	4 (2–5.8)	3 (1–5)	0.0078
Comorbidities, *n* (%)			
Diabetes mellitus type 2	59 (33.91%)	33 (16.18%)	0.0001
Arterial hypertension	126 (72.41%)	90 (44.12%)	0.0001
Dyslipidemia	54 (31.03%)	36 (17.65%)	0.0025
Chronic obstructive pulmonary disease	21 (12.07%)	9 (4.41%)	0.0072
Gastritis/gastroesophageal reflux disease	18 (10.34%)	19 (9.31%)	0.8624
Cardiovascular diseases	46 (26.44%)	48 (23.53%)	0.5515
Chronic renal insufficiency	13 (7.47%)	9 (4.41%)	0.2707
Peripheral vascular disease	11 (6.32%)	12 (5.88%)	1.0000
Neurological diseases	29 (16.67%)	42 (20.59%)	0.3570
Metabolic syndrome	110 (63.22%)	47 (23.04%)	<0.0001
MetS median score	3 (2–4)	2 (1–2)	<0.0001
Chronic medications			
Beta blockers	47 (27.01%)	48 (23.53%)	0.4761
ACE inhibitors	78 (44.83%)	59 (28.92%)	0.0018
Other antihypertensive drugs	75 (43.10%)	55 (29.96%)	0.0011
Statins	44 (25.29%)	24 (11.76%)	0.0007
Metformin	34 (19.54%)	18 (8.82%)	0.0028
Another oral anti-diabetic	37 (21.26%)	18 (8.82%)	0.0007
Insulin	25 (14.37%)	21 (10.29%)	0.0465
Antiplatelet agent	25 (14.37%)	21 (10.29%)	0.2697
Duration of symptom onset to hospital admission	4 (2–6)	4 (2–7)	0.2722
Sepsis severity scores			
SIRS	2 (2–3)	2 (2–3)	0.9919
SOFA	3 (2–5)	3 (2–4)	0.0159
APACHE II	13 (9–20)	12 (8–18)	0.0766
Liver related scores			
FAST score	0.31 (0.15–0.52)	0.1 (0.04–0.27)	<0.0001
APRI score	0.46 (0.23–1.1)	0.36 (0.22–0.73)	0.0429
FIB-4 score	2.1 (1.2–3.4)	1.6 (0.91–2.7)	0.0056
NAFLD score	0.43 (−0.62–2.1)	−0.48 (−1.9–1.1)	<0.0001
Infection source			
Pneumonia	39 (22.41%)	48 (23.41)	0.3597
Skin and soft tissue	32 (18.39%)	21 (10.24%)	
Gastrointestinal tract	28 (16.09%)	28 (13.66%)	
Urinary tract	26 (14.94%)	45 (21.95%)	
Other	20 (11.49%)	24 (11.71%)	
Unknown	15 (8.62%)	21 (10.24%)	
Etiology identified	86 (49.43%)	118 (57.84)	0.1017

* Fisher exact or Mann–Whitney U test, as appropriate.

**Table 2:** Clinical outcomes of sepsis in patients with and without SLD.

	SLD (*n* = 174)	Non-SLD (*n* = 204)	*p*-Value *
Primary outcomes			
In-hospital mortality	32 (18.39%)	20 (9.80%)	0.0157
Time to death from hospital admission	7 (4–17)	18 (10–28)	0.0020
48-h mortality	4 (2.30%)	0 (0%)	0.0295
7-day mortality	17 (9.77%)	2 (0.98%)	<0.0001
28-day mortality	27 (15.52%)	17 (8.33%)	0.0300
Secondary outcomes			
Length of hospital stay	10 (7–17)	12 (7–23)	0.1233
ICU admission	64 (36.78%)	62 (30.39%)	0.1890
Vasopressor therapy	53 (30.46%)	47 (23.04%)	0.1031
Duration of vasopressor therapy	3 (1–7)	2 (1–6)	0.6191
Moderate/severe ARDS	40 (22.99%)	32 (15.69%)	0.0715
Invasive mechanical ventilation	51 (29.31%)	37 (18.14%)	0.0104
Duration of IMV	7 (2–14)	6 (1–16)	0.8038
Acute kidney injury	46 (29.31%)	36 (17.65%)	0.0388
Continuous renal replacement therapy (CRRT)	28 (16.09%)	18 (8.82%)	0.0312
Duration of CRRT	3.0 (2–8)	2 (1–16)	0.3908
Nosocomial infections	30 (17.24%)	24 (11.76%)	0.1294
Acute heart failure	16 (9.20%)	14 (6.86%)	0.4030

* Fisher exact or Mann–Whitney U test, as appropriate.

**Table 3:** Univariable and Cox proportional regression analysis of factors associated with mortality in patients with sepsis.

	Univariable Analysis		Cox Proportional Regression Analysis	
	Odds Ratio (95% CI)	*p*-Value	Hazard Ratio (95% CI)	*p*-Value
Age ≥ 60 years	1.07 (0.55–2.06)	0.8214		
Male sex	0.862 (0.64–2.12)	0.6556		
CAP ≥ 270 dB/m	1.173 (1.081–3.597)	0.0223		
Liver stiffness ≥ 6.6 kPa	2.013 (1.115–3.577)	0.0185		
SOFA ≥ 5	4.271 (2.27–7.69)	<0.0001	2.611 (1.493–4.581)	0.0007
T2DM	2.19 (1.20–4.02)	0.0053		
SLD	2.08 (1.15–3.82)	0.0170	2.824 (1.40–5.71)	0.0276
Procalcitonin ≥ 3.0 µg/L	2.456 (1.37–4.52)	0.0027		
Lactate ≥ 2.5 mmol/L	5.64 (2.96–10.40)	<0.0001	2.599 (1.133–6.259)	0.0265
Blood urea nitrogen ≥ 9.0 mmol/L	2.018 (1.12–3.69)	0.0181		
Creatinine ≥ 115 µmol/L	3.075 (1.66–5.43)	0.0001		
eGFR ≤ 50 mL/min/1.73 m^2^	2.75 (1.49–5.09)	0.0006		
LDH ≥ 260 IU/L	2.931 (1.62–5.43)	0.0003		
Albumin ≤ 30 g/L	2.156 (1.18–3.96)	0.0113		
INR ≥ 1.3	6.115 (3.24–11.48)	<0.0001	2.225 (1.13–4.42)	<0.0001
D-dimer ≥ 4.0 mg/L	4.100 (2.19–7.46)	<0.0001		
Cholesterol ≤ 2.8 mmol/L	5.149 (2.07–11.88)	<0.0001		
Triglycerides ≤ 1.7 mmol/L	4.113 (1.68–9.35)	<0.0001		
HDL ≤ 0.6 mmol/L	2.750 (1.17–6.26)	0.0165		
NAFLD fibrosis score ≥ 1.3	5.545 (2.97–9.97)	<0.0001		
FIB-4 score ≥ 3.0	4.558 (2.46–8.42)	<0.0001		
FAST score ≥ 0.35	3.659 (2.00–6.69)	<0.0001	2.244 (1.240–4.124)	0.0081
Respiratory tract origin	2.19 (1.20–4.13)	0.0122		
Non-urinary tract origin	10.12 (1.74–10.46)	0.0055	1.529 (1.037–2.217)	0.028
ICU admission	14.95 (6.593–29.59)	<0.001		
Renal insufficiency	9.078 (4.684–16.72)	<0.001	5.586 (2.687–12.02)	<0.0001
CRRT	22.99 (11.05–48.82)	<0.001		
IMV	17.02 (8.542–33.38)	<0.001	3.951 (1.065–13.85)	0.0374
ARDS	8.328 (4.474–15.54)	<0.001		
Shock > 24 h	31.79 (13.87–76.94)	<0.001		
Nosocomial infections	5.397 (2.747–10.73)	<0.001	3.839 (1.503–10.58)	0.0066

Odds ratios (OR) and hazard ratios (HR) with corresponding 95% confidence intervals (95% CI) are shown. Shown are only factors significantly associated with mortality, while detailed data including factors which did not differ between survivors and non-survivors are provided in Supplementary Table S3.

## Data Availability

The datasets generated during and/or analyzed during the current study are available from the corresponding author upon reasonable request.

## Supplementary Materials

The following supporting information can be downloaded at: https://www.mdpi.com/article/10.3390/jcm13030798/s1, Table S1: Etiology of sepsis; Table S2: Laboratory findings on hospital admission; Table S3: The differences in baseline clinical, laboratory and microbiological characteristics between survivors and non-survivors.
