# Supplementary material for: Steatotic Liver Disease and Sepsis Outcomes—A Prospective Cohort Study (SepsisFAT)
Source: J Clin Med. 2024 Jan 30;13(3):798. doi: 10.3390/jcm13030798 (PMC10856507; doi:10.3390/jcm13030798)
Supplement: Supplementary file 1 [file jcm-13-00798-s001.zip › jcm-2826392-supplementary.pdf]

Supplementary Table S1. Etiology of sepsis

	NAFLD (%)	non-NAFLD (%)
**Positive blood cultures (Gram stain)**	50 (28.74%)	54 (26.47%)
Escherichia coli (G-)	11 (22.00%)	13 (24.07%)
methicillin-susceptible *Staphylococcus aureus* (G+)	8 (16.00%)	7 (12.96%)
Streptococcus pneumoniae (G+)	6 (12.00%)	9 (16.67%)
group A β-hemolytic *streptococci* (G+)	4 (8.00%)	5 (9.26%)
group B β-hemolytic *streptococci* (G+)	3 (6.00%)	0 (0.00%)
Streptococcus spp. (G+)	3 (6.00%)	2 (3.70%)
Klebsiella pneumoniae (G-)	3 (6.00%)	3 (5.56%)
Listeria monocytogenes (G+)	2 (4.00%)	1 (1.85%)
Pseudomonas aeruginosa (G-)	2 (4.00%)	2 (3.70%)
Salmonella spp. (G-)	2 (4.00%)	2 (3.70%)
Bacteroides spp. (G-)	2 (4.00%)	1 (1.85%)
Fusobacterium nucleatum (G-)	1 (2.00%)	2 (3.70%)
Enterococcus spp. (G+)	1 (2.00%)	3 (5.56%)
Kingella kingae (G-)	1(2.00%)	0 (0.00%)
Enterobacter spp. (G-)	1 (2.00%)	2 (3.70%)
Citrobacter spp. (G-)	0 (0.00%)	2 (3.70%)
**Positive urine cultures**	38 (21.84%)	55 (26.96%)
**Positive stool cultures**	21 (12.07%)	23 (11.27%)
**Positive respiratory cultures**	12 (6.90%)	12 (5.88%)
**Other**	23 (13.22%)	32 (15.69%)
**Etiology unidentified**	88 (50.57%)	86 (42.16%)
**Etiology identified**	86 (49.43%)	118 (57.84%)

Supplementary Table S2. Laboratory findings on hospital admission

Laboratory findings	Reference range	NAFLD	non-NAFLD	p-Value
C-reactive protein, mg/L	< 5	206 (118-291)	198 (88-280)	0.4614
Procalcitonin, μg/L	< 0.05	0.83 (0.24-8.2)	0.9 (0.21-5.2)	0.4787
Lactate, mmol/L	< 2	2 (1.2-3.1)	1.9 (1.3-3.1)	0.6674
White blood cells, $x10^9$/L	3.4 – 9.7	13 (8.8-16.0)	13 (9-18)	0.4939
ANC, $x10^9$/L	2.06 – 6.49	10 (6.5-16.0)	9.7 (6.4-14.0)	0.4128
ALC, $x10^9$/L	1.19 – 3.35	0.84 (0.51-1.2)	0.78 (0.46-1.5)	0.7017
Ne/Ly ratio	1-3	12 (6.5-26.0)	12 (5.4-24.0)	0.5699
Hemoglobin, g/L	138 -175	127 (113-137)	122 (105-133)	0.0137
Hematocrit	0.356 – 0.470	0.38 (0.34-0.41)	0.36 (0.32-0.4)	0.0202
MCV	83.0 – 97.2	89 (84-92)	88 (85-91)	0.7432
Platelets, $x10^9$/L	158 - 424	210 (153-269)	216 (159-285)	0.5032
Plt/Ly ratio	90 - 210	240 (164-401)	232 (144-375)	0.4232
Fibrinogen, g/L	1.8 – 3.5	6 (4.9-7.9)	5.9 (4.9-8.1)	0.8013
International normalized ratio (INR)	0.8–1.2	1.1 (0.97-1.2)	1.1 (0.97-1.2)	0.9493
D-dimer, mg/L	<0.50	2 (1-4.2)	1.7 (1-4.2)	0.3881
Glucose, mmol/L	4.4 – 6.4	7.6 (6.5-9.8)	6.7 (5.8-8.1)	<0.0001
Blood urea nitrogen, mmol/L	2.8 – 8.3	8 (5.2-12)	6.7 (5.1-11)	0.1904
Creatinine, μmol/L	64 – 104	93 (71-146)	84 (63-118)	0.0429
eGFR (MDRD)	>90	64 (36-86)	69 (45-94)	0.0314
Na, mmol/L	137 - 146	139 (135-141)	138 (136-142)	0.8687
Potassium, mmol/L	3.9 – 5.1	3.8 (3.5-4.3)	3.9 (3.6-4.2)	0.2416
Total bilirubin, μmol/L	3 -20	12 (8-18)	12 (7.7-17)	0.3111
Aspartate aminotransferase, U/L	8 – 38	33 (23-60)	26 (18-48)	0.0002
Alanine aminotransferase, U/L	10 – 48	33 (19-55)	24 (15-45)	0.0069
Gamma-glutamyl transferase, U/L	11 – 55	54 (29-116)	34 (20-67)	<0.0001
Alkaline phosphatase, U/L	60 - 142	76 (60-103)	80 (63-102)	0.4812
Lactate dehydrogenase, U/L	<241	204 (176-274)	198 (161-262)	0.1101
Troponin T, ng/L	< 14	0.029 (0.015-0.064)	0.024 (0.012-0.053)	0.2234
Total serum proteins, g/L	66 – 81	61 (56-67)	61 (56-67)	0.9688
Serum albumins, g/L	41 – 51	32 (29-37)	32 (28-38)	0.8048
Triglycerides, mmol/l	<1.7	1.9 (1.4-2.9)	1.4 (1.1-2)	<0.0001
Cholesterol, mmol/L	< 5.0	3.9 (3.2-4.8)	3.8 (2.9-4.7)	0.3802
LDL, mmol/L	< 3,0	2.4 (1.9-3.1)	2.3 (1.6-2.9)	0.2125
HDL, mmol/L	>1.0	0.7 (0.6-0.9)	0.8 (0.6-1)	0.1167
**Liver related scores**				
FAST score		0.31 (0.15-0.52)	0.1 (0.04-0.27)	<0.0001
FAST score >0.35		77 (44.25%)	39 (19.12%)	<0.0001
APRI score		0.46 (0.23-1.1)	0.36 (0.22-0.73)	0.0429
FIB-4 score		2.1 (1.2-3.4)	1.6 (0.91-2.7)	0.0056
NAFLD score		0.43 (-0.62-2.1)	-0.48 (-1.9-1.1)	<0.0001
NAFLD score (<1.4)		23 (13.22%)	63 (30.88%)	0.0002

Supplementary Table S3. The differences in baseline clinical, laboratory and microbiological characteristics between survivors and non-survivors

	Non-survivors	Survivors	p-Value
Age, years, median (IQR)	69 (55-79)	67 (54-78)	0.4871
Male sex, n (%)	24 (46.15%)	163 (49.85%)	0.6563
Body mass index, kg/m2	27 (25-30)	27 (24-31)	0.3563
Waist-hip ratio	1 (0.93-1)	0.96 (0.9-1)	0.1594
Waist-height ratio	1.8 (1.7-2.1)	1.9 (1.7-2.1)	0.9092
Controlled Attenuation Parameter (dB/m)	270 (224-300)	239 (196-293)	0.0107
Liver stiffness (kPa)	6.6 (5.1-8.7)	5.7 (4.1-8.2)	0.037
Smoker	8 (15.38%)	67 (20.49%)	0.4577
Moderate alcohol consumption	4 (7.69%)	37 (11.31%)	0.6302
Charlson comorbidity index	3.5 (2-6.8)	3 (2-5)	0.099
**Comorbidities, n (%)**			
NAFLD	32 (65.54%)	142 (43.43%)	0.0167
Diabetes mellitus type 2	21 (40.38%)	71 (21.71%)	0.0052
Arterial hypertension	33 (63.46%)	183 (55.96%)	0.3364
Dyslipidemia	15 (28.85%)	75 (22.94%)	0.381
Chronic obstructive pulmonary disease	5 (9.62%)	25 (7.65%)	0.5838
Gastritis / GERD	7 (13.46%)	30 (9.17%)	0.319
Cardiovascular diseases	13 (25.00%)	81 (24.77%)	>0.9999
Chronic renal insufficiency	5 (9.62%)	17 (5.20%)	0.2041
Peripheral vascular disease	3 (5.77%)	20 (6.12%)	>0.9999
Neurological diseases	12 (23.08%)	59 (18.04%)	0.4433
Metabolic syndrome	20 (38.46%)	137 (41.90%)	0.7621
MetS median score	2 (1-3)	2 (1-3)	0.17
**Sepsis severity scores**			
SOFA	5 (4-7)	2 (1-4)	<0.0001
APACHE II	18 (10-24)	12 (8-18)	0.0006
**Liver related scores**			
FAST score	0.37 (0.23-0.62)	0.19 (0.06-0.39)	<0.0001
FIB-4 score	3 (1.5-6.8)	1.7 (1.1-2.9)	<0.0001
NAFLD score	1.3 (-0.08-3.3)	-0.05 (-1.2-1.3)	0.0003
**Infection source**			
Pneumonia	19 (36.54%)	68 (20.80%)	0.0082
Skin and soft tissue	4 (7.69%)	49 (14.98%)	
Gastrointestinal tract	9 (17.31%)	47 (14.37%)	
Urinary tract infection	1 (1.92%)	70 (21.41%)	
Other	15 (26.92%)	62 (18.96%)	
Unknown	5 (9.62%)	31 (9.48%)	
**Laboratory findings on admission**			
C-reactive protein, mg/L	203 (134-305)	197 (86-280)	0.3003
Procalcitonin, µg/L	3.3 (0.31-50)	0.73 (0.22-4.2)	0.007
Lactate, mmol/L	2.6 (1.9-4.2)	1.9 (1.1-3)	0.0003
White blood cells, x10^9/L	12 (7.8-15)	13 (8.9-17)	0.3829
ANC	9.9 (6.9-13)	10 (6.4-15)	0.951
ALC	0.67 (0.31-1.1)	0.85 (0.53-1.3)	0.0318
Ne/Ly ratio	15 (6.6-35)	12 (6-24)	0.1162

Hemoglobin, g/L	125 (101-136)	125 (108-136)	0.3704
Hematocrit, L/L	0.37 (0.3-0.41)	0.38 (0.33-0.41)	0.3662
Platelets, x10^9/L	191 (130-263)	214 (160-282)	0.0861
Plt/Ly ratio	245 (144-543)	232 (160-368)	0.2698
Fibrinogen, g/L	5.7 (4.6-7)	6 (4.9-8.1)	0.1135
International normalized ratio (INR)	1.3 (1.1-1.7)	1 (0.96-1.2)	<0.0001
D-dimer, mg/L	4.2 (2.7-4.3)	1.8 (0.93-3.6)	<0.0001
Glucose, mmol/L	8.5 (6.4-11)	7.1 (6-8.7)	0.0515
Blood urea nitrogen, mmol/L	9.3 (7.2-15)	7 (5.1-11)	0.0027
Creatinine, µmol/L	116 (72-201)	85 (66-118)	0.0108
eGFR (MDRD)	52 (27-78)	68 (45-92)	0.0038
Total bilirubin, µmol/L	15 (11-24)	12 (7.9-17)	0.0019
Aspartate aminotransferase, U/L	41 (25-142)	27 (19-51)	0.0008
Alanine aminotransferase, U/L	35 (14-79)	27 (16-47)	0.3546
Gamma-glutamyl transferase, U/L	46 (22-97)	39 (22-79)	0.4206
Lactate dehydrogenase, U/L	268 (182-442)	197 (167-251)	0.0008
Serum albumins, g/L	30 (22-33)	33 (29-37)	<0.0001
Triglycerides, mmol/l	2.2 (1.4-2.9)	1.7 (1.2-2.2)	0.0856
Cholesterol, mmol/L	2.8 (2-3.7)	3.9 (3.2-4.8)	0.0002
LDL, mmol/L	1.7 (1.4-2.5)	2.4 (1.9-3.1)	0.003
HDL, mmol/L	0.6 (0.4-0.8)	0.8 (0.7-1)	0.0016
**Positive blood cultures**	20 (36.84%)	84 (25.69%)	0.0627
**Clinical course and treatment**			
ICU admission	43 (82.69%)	83 (25.38%)	<0.0001
Vasopressors need	45 (86.54%)	55 (16.82%)	<0.0001
Moderate/severe ARDS	29 (55.77%)	43 (13.15%)	<0.0001
Invasive mechanical ventilation	39 (75.00%)	49 (14.98%)	<0.0001
Acute kidney injury	32 (61.54%)	49 (14.98%)	<0.0001
Continuous renal replacement therapy	29 (55.77%)	17 (5.20%)	<0.0001
Nosocomial infections	17 (32.69%)	27 (8.26%)	<0.0001
